# 

**Published:** 1848-10

**Authors:** 


					VOL, IX.
.8.
lo. 1.
R I
OP
Science.
P U BLIS H ED
.
PUBLISHED UtfDEk THE AUWICE^OF THE
'# ' J
LY
a socfrts of ?tntai ? _
EDITORS
HARRIS, M. D., D. D. S.
OTT, M. BtfO. D. 1*1
H. DWINELLE, D. D.lf
?. D. S.,7 Gower-st. Bedford Square,
BALTIMORE:
& B E k* KY t
MJL
S*d
|SOO per anua
This Periodica] weighs 9 ounces.

				

